# Event and Comment

**Published:** 1908-07-15

**Authors:** 


### DENTAL REGISTER.

**Vol. LXII. July 15, 1908. No. 7.**

### EVENT AND COMMENT.

### Indiana Semi-Centennial Celebration.

Nearly five hundred dentists from far and near met at Indianapolis June fourth to sixth to celebrate the fiftieth birthday of the State Society. It was a significant affair in several ways, but most peculiar in view of the fact that only a small part of the most excellent program was contributed by Indiana dentists. Almost the entire program in essays and clinics was contributed by dentists from Ohio, Illinois, Kentucky, Michigan, Missouri, Kansas, Iowa, Pennsylvania and Minnesota. It was a most cordial and fraternal gathering, in which every visitor felt that he was bound to pay homage to the host in a manner that would illustrate what had been accomplished in the life time of this society, which is the third oldest dental society in existence. The entertainment by Indiana to the swarm of guests was all that any one could desire. The place of meeting and the arrangements were most excellent, and everyone who came went away satisfied. The papers and clinics were of the highest order and set forth in a very satisfactory manner the present attainments in technical dentistry especially. The one thing that impressed us most was the high professional and fraternal feeling that was manifested. There were no disagreeable discussions that sometimes mar ones pleasure and detract from the value of the meeting because some egotistical or dyspeptic individual consumes more than his share of the time in self laudation, or in endeavoring to detract from the value of some ones earnest work. But how could anything but a love feast be expected in a city where Hunt and Byram live and with such visitors and participants as Black, Brophy, Hartzel, Fletcher, Rhein, Patterson, Wright, Custer, Thorpe and a host of other good fellows.

### The Michigan State Meeting.

In the matter of novelty this society carried off the prize this year by holding its annual convention on a steamboat. A four days trip on a finely equipped and commodious steamer, well officered and bountifully stocked with good things to eat, which were most bountifully served at regular intervals during the entire trip made this occasion one that will long be remembered by all who took the trip as one of the most delightful outings imaginable. The boat was commodious and comfortable in every way, and the weather was simply perfect. With plenty of music and good cheer it was simply ideal. The boat left the dock at Detroit as appointed and made the trip as scheduled to Lake Superior and returned without a single accident of any kind and on time to the minute. There certainly never was a better behaved and more thoroughly satisfied body of excursionists on such a trip, and it was the universal expression of every one that they had experienced a most delightful trip and were so enthusiastic that it is now the talk that it shall be a feature of each annual meeting. The program was carried on under the strict discipline of the President without a hitch and every meeting was well attended. One day was spent at Sault Ste. Marie where the general clinics were given in the large room of the City Hall. As it was not practicable to give some of the clinics on the boat because of proper facilities being lacking. The meeting at the Soo attracted a considerable number of North Peninsula dentists and also quite a number from Canada. Some of whom were so pleased with the meeting that they returned to Mackinac Island on the boat. The program was good and well carried out. One novel feature was a presentation by the Second District Society of a special program of two papers and twelve clinics the last day and evening on the boat. It is hoped next year to have the several District Societies each contribute their work at special sessions. It was a most unique and profitable meeting in every way and we hope it may be duplicated in the near future, especially for the benefit of some who did not go because they predicted a failure, or that the weather would be bad, or for some other reason best known to themselves. It was a fine occasion for social and fraternal intercourse and many acquaintances were made and friendships formed that would not have occurred under ordinary circumstances.

### The Fifty-First Annual Meeting of the Northern Ohio Dental Association.

The Association met May 26, 27 and 28 as advertised at Canton. We were anticipating a very enjoyable meeting, and realized our expectations. Canton is a beautiful little city and it is doubtful if any city in the State is so well equipped to care for so large an association. The city's Auditorium was well adapted to the needs of the meeting; and, gratuitously provided. The work of the executive committee was well done.

All the essayists fulfilled their parts save one. Owing to a painful but not serious automobile accident, Dr. Harlan, of New York, was unable to be present. Nearly all the fifty-two clinicians responded. However, there were a few absent; probably some of the absent ones were excusable, but with others, there is always a question. Some men are prone to accept an invitation to give a clinic, when, apparently they have no intention of meeting the obligation. Why is it? Henceforth this evil should be reduced to a minimum, as one of the amendments to the constitution, adopted this year, makes the executive committee elective, one member being elected each year for a term of three years. This change will make the committee practically continuous; and one of their assumed duties should be to see that the society is not imposed upon a second time by those men who accept a position upon the program for an unworthy purpose.

Another important change in the constitution is the creating of an annual due of one dollar. In the fifty-one years of the life of this society there has been no dues, and only two assessments levied. The society's meetings have always been open to everybody, of course membership limited to ethical practitioners. An effort was made to still retain this feature of no dues but making a charge of one dollar for attending the clinics. This plan would probably raise more funds than the membership dues and still retain the missionary feature so long associated with the society. All acquainted with the trend of society work were convinced that a reliable source of revenue is a necessity, only differed in the method of its production. Some of the arguments used against the clinic fee method were indeed amusing to say the least. One was that it was an innovation, that no other society used that means of raising funds; as though there were something divine in stereotyped methods. Oh, that men could contemplate ideas rather than forms! Another argument was: That it favored the charlatans in that by paying their dollar they had all the advantages of the members. That is not so. Are there no advantages of membership except attendance upon clinics? We hold that as it is now the so-called charlatan gets all he wants and saves his dollar; unless everybody is excluded from the clinics except members who have paid their dues. We trust the Northern Ohio Dental Association will never smirch her royal robes by any such quack methods. We know that other societies make this mistake, we believe they are victims of precedent and have not contemplated the question sufficiently to draw logical conclusions. The dental profession claims to be a liberal profession and decries closed doors, secret methods, nostrums, et cetera. The idea of our profession is that it exists by virtue of the relief it affords to suffering humanity, that the individual or a coterie of individuals have no right to exclusively appropriate professional lore, that what is for the benefit of humanity belongs to the public. Is not an association an unpardonable quack when it closes its doors and admits only the elect? What is a charlatan and a quack anyway? Our superficial professional definition is: One who advertises; while the real meaning is: An ignorant pretender, one who imposes upon the public. When a man complies with the laws of the State and becomes a legal practitioner he cannot *legally* be classed as ignorant and a pretender. If he chooses to use paid advertisements in the public prints he has done nothing *illegal* nor *immoral; but he has* shown poor taste and a lack of refinement. Charlatans may, or they may not use the public print. It is impossible to have a code of ethics that will keep all the charlatans out of dental societies, for we should remember: "There is no man so good, who, were he to submit all his thoughts and actions to the laws, would not deserve hanging ten times in his life." We should not forget the spirit of the code of ethics, which is, to be kindly and generously disposed toward a weaker brother. If the motives of the Northern Ohio Dental Society, for the past fifty-one years have been good and wise, we should be very chary about reversing our policy from an open to a closed one. There are many reasons why good and desirable men hesitate to become members of dental societies, who, if but properly fostered, may eventually become active and worthy co-laborers. These men are willing to help pay the expenses of the society, but may not be able, for intrinsic and extrinsic reasons, to assume membership. By repelling these men the society may do untold harm. This class of dentists is a very large one, consisting of the majority of the young men just starting in practice, a few older ones having diffident natures, and not a few men having neighbors, who are members of the society and obsessed of the green eyed monster. We trust our venerable and beloved society will never adopt the closed door policy.

There was a feature of this meeting which pleased us very much. A few years ago one of our noted teachers of operative dentistry stated in a discussion in a meeting of the Dental Pedagogics Society that he questioned whether our modern methods of teaching were producing the quality of dentists that were created by the office pupilage method. As we listened to the dissertations of the invited guests Drs. C. R. Turner, F. B. Noyes and Mary Hartzell, we could have only optimistic thoughts for the future of our profession. When we consider the quality of the work these young people are doing, imagination only can picture to us the fruits of their matured mentalities twenty-five years hence. We are confident there are many more of the rising generation that will be bright stars in the dental firmament.—G. H. Wilson, in *Dentists Magazine*.

### The Section or Stomatology, A. M. A.

The annual meeting of this section was held in Chicago June 2 to 5 in connection with the regular annual meeting of the A. M. A. A most excellent program was provided, but as is usual in this section there were so few in attendance that the discussions were not so valuable as they should have been. Although there were ten thousand physicians in attendance at this meeting and there are more than one thousand dentists in Chicago and its immediate vicinity, there were at no session of the dental section more than thirty or forty dentists, and at most of the sessions twelve or fifteen only would show up. Why is it that dentists do not attend or identify themselves with this work? Is it the fault of the management, or is it due to the idea of the dentists that they don't wish to become an apendage to the medical kite? There must be a reason, and if it is found that the connection of dentists with doctors is incompatible or even undesirable it seems to us that this Section should be abandoned. Or if it is to be maintained it should be supported by a respectable and representative attendance, otherwise it is of no credit to our profession and even may subject us to the contempt of the medical profession.

